# Ciprofloxacin and Graphene Oxide Combination—New Face of a Known Drug

**DOI:** 10.3390/ma13194224

**Published:** 2020-09-23

**Authors:** Karolina Matulewicz, Łukasz Kaźmierski, Marek Wiśniewski, Szymon Roszkowski, Krzysztof Roszkowski, Oliwia Kowalczyk, Archi Roy, Bartosz Tylkowski, Anna Bajek

**Affiliations:** 1 Chair of Urology, Department of Tissue Engineering, Collegium Medicum, Nicolaus Copernicus University, Karlowicza str. 24, 85-092 Bydgoszcz, Poland; kazmierski.lkk@gmail.com (Ł.K.); a_bajek@wp.pl (A.B.); 2 Department of Oncology, Collegium Medicum, Nicolaus Copernicus University, Lukasiewicza str. 1, 85-821 Bydgoszcz, Poland; roszkowskik@cm.umk.pl; 3 Department of Chemistry of Materials Adsorption and Catalysis, Nicolaus Copernicus University, Gagarina str. 7, 87-100 Torun, Poland; Marek.Wisniewski@umk.pl; 4 Faculty of Agronomy and Bioengineering, Poznan of Life Sciences, Wojska Polskiego str. 28, 60-637 Poznan, Poland; roszkowski.sz11@gmail.com; 5 Research and Education Unit for Communication in Healthcare, Department of Cardiac Surgery, Ludwik Rydygier Collegium Medicum in Bydgoszcz Nicolaus Copernicus University in Torun, M. Curie Sklodowskiej St. 9, 85-094 Bydgoszcz, Poland; oliwia.kowalczyk@cm.umk.pl; 6 Departament d’Enginyeria Química, Universitat Rovira i Virgili, Avda. Països Catalans, 26. Ed. E4. (C. Sescelades), 43007 Tarragona, Spain; archiroy07@gmail.com; 7 Eurecat, Centre Tecnològic de Catalunya, C/Marcellí Domingo s/n, 43007 Tarragona, Spain; bartosz.tylkowski@eurecat.org

**Keywords:** carbon-based materials, graphene oxide, drug modification

## Abstract

Drug modification with nanomaterials is a new trend in pharmaceutical studies and shows promising results, especially considering carbon-based solutions. Graphene and its derivatives have attracted much research interest for their potential applications in biomedical areas as drug modifiers. The following work is a comprehensive study regarding the toxicity of ciprofloxacin (CIP) modified by graphene oxide (GO). The influence on the morphology, viability, cell death pathway and proliferation of T24 and 786-0 cells was studied. The results show that ciprofloxacin modified with graphene oxide (CGO) shows the highest increase in cytotoxic potential, especially in the case of T24 cells. We discovered a clear connection between CIP modification with GO and the increase in its apoptotic potential. Our results show that drug modification with carbon-based nanomaterials might be a promising strategy to improve the qualities of existing drugs. Nevertheless, it is important to remember that cytotoxicity effects are highly dependent on dose and nanomaterial size. It is necessary to conduct further research to determine the optimal dose of GO for drug modification.

## 1. Introduction

According to a recently released report by the International Agency for Research on Cancer from the World Health Organization (WHO), the global cancer burden was estimated at 18.1 million new cases and 9.6 million deaths in 2018 [1]. Among the genitourinary system cancers, renal cell carcinoma (RCC) and transitional cell carcinoma (TCC) are the ones which recently attracted the attention of oncologists and scientists due to insufficient therapy efficiency (high systematic toxicity), high mortality rates, and a constant increase in the number of patients diagnosed with such types of cancer [2]. Currently, most of the patients with these cancer types have been treated with chemotherapy, which is considered the gold standard treatment [3]. Unfortunately, its success is often hampered by multidrug resistance (MDR) [4], active removal of drugs from the cells [5], and poor bioavailability and non-specific targeting [5]. For this reason, oncologists have called for fundamental studies and new upstream technological innovations to respond sustainably, efficiently, and safely to current and future patients’ needs [6]. Furthermore, considering the occasional lack of efficiency of treatment methods such as traditional chemotherapy, radiotherapy, or surgery, the oncology society has demanded from scientists solutions in the form of the development of new materials which allow drug delivery with the final goal of maximizing therapeutic efficacy [7]. Recently, multiple studies have documented that modified-drug therapy is emerging as a promising approach for the treatment of cancer. Moreover, nowadays, scientists’ and technologists’ expanding knowledge concerning nanotechnology provides a unique opportunity for the combination of exceptional properties of nanoparticles and nanomaterials for precise introduction of the active compound into the tumor [8]. Inspired by a released review in the Nature publishing platform [9] concerning the outstanding properties of graphene-based smart materials, we unquestionably decided to follow the current research trends and to employ graphene oxide (GO) as a promising strategy to enhance the therapeutic index for ciprofloxacin (CIP) as an anticancer agent for genitourinary system treatment. CIP is an organic compound, included in the second generation of fluoroquinolones, which is characterized by good tissue penetration and favorable pharmacokinetic parameters [10]. For a long time, ciprofloxacin was thought to affect only bacterial cells [10,11,12], but currently, it is widely known that this drug can significantly reduce the viability of human cancer cells [13,14,15]. We believe that an innovative transformation of traditional CIP to GO-CIP nanoplatform could introduce a new treatment perspective with a significant effect on the reduction of side effects in normal cells, enhancement of their anticancer activity as well as the increase of their stability. Indeed, GO possesses the ability to accumulate in tissues, including cancer tissues [16,17], and due to its affinity to lower pH levels [18], GO-drug modified nanoplatforms could increase the chances of targeting a specific region of interest, being the cancer tumor, while decreasing systemic toxicity.

The aim of this study was to perform a comprehensive in vitro analysis of the CIP-GO nano-platforms for potential RCC and TCC treatment.

## 2. Materials and Methods

### 2.1. Graphene Oxide Characterization

The High Resolution Transmission Electron Microscopy (HRTEM) images were taken using a transmission electron microscope F20X-TWIN (FEI-Tecnai) operated at 200 kV. The drop of sample solution was placed on a Cu grid coated with an ultrathin amorphous carbon film and then dried under ambient conditions.

The XRD measurements were performed using Philips XPERT Proθ-2θ with X’Celerator Scientific detector with CuKα1 radiation. Data were collected from 5 ≤ 2θ/≤120°, with a step size of 0.0084° 2θ and at a scanning rate of 0.02°/min.

For the Fourier transform infrared (FTIR) measurements, spectra were accomplished by Mattson Genesis II infrared spectrophotometer (Mattson, Foster City, CA, USA) using transmission mode techniques in the frequency range 400–6000 cm^−1^ for the GO self-supporting paper.

For the Raman measurements, the nonpolarized spectra of carbon structures were investigated in the spectral range of 60–4500 cm^−1^. The spectra were recorded in the backscattering geometry using SENTERRA micro-Raman system (Bruker Optik). As an excitation light, we used a green laser operating at 532 nm. The laser beam was tightly focused on the sample surface through a 50× microscope objective. To prevent any damage of the sample, the excitation power was fixed at 0.2 mW. The resolution was 9–15 cm^−1^, and CCD temperature of 223 K, laser spot of around 2 μm, and total integration time of 100 s (50 × 2 s) were used. The position of the microscope objective with respect to the sample was piezoelectrically controlled in XY position [19,20,21].

### 2.2. Preparation of Ciprofloxacin Solution

In order to obtain the concentrated solution of ciprofloxacin (CIP), the powdered drug (Sigma-Aldrich, Saint Louis, MO, USA) was dissolved in slightly acidic solution pH (4.7) to increase solubility. The PBS was titrated with HCl then filtered using a 0.22 syringe filter (Merck Millipore, Burlington, MA, USA). Ciprofloxacin concentrations in culture medium at the range of 10–1000 μM were used in the experiment. 

### 2.3. Preparation of Graphene Oxide

The detailed synthesis description and characteristic of graphene oxide (GO) was reported previously [19,20,21]. Briefly, the material was synthesized using the modified Hummers method [22]. A mixture of concentrated H_2_SO_4_/H_3_PO_4_ (360:40 mL) was poured onto graphite flakes (3 g). KMnO_4_ (18 g) was slowly added to the mixture, producing slightly exothermic conditions (35–40 °C). After 24 h of continuous stirring, 400 mL of water and 5 mL of 30% H_2_O_2_ were added, producing a bright yellow sol. The mixture was centrifuged at 15,000× *g* for 30 min, and the supernatant was removed. The remaining material, in gel form, was next washed successively with water and ethanol and centrifuged repeatedly (several times) to adjust the pH 6.

Before using the graphene oxide, it was diluted in deionized water and treated with ultrasound for 60 min to obtain a liquid GO solution. GO controls were used in the experiment in concentrations ranging from 10 to 1000 μg/mL.

### 2.4. Preparation of Ciprofloxacin and Graphene Combination

Modified ciprofloxacin was prepared by mixing the solutions, maintaining the mass ratio of 1:1 and 1:2 CIP to GO. Finally, only a ratio of 1:1 was chosen for the in vitro studies. 

### 2.5. pH Test of Cell Culture Medium

To eliminate the possibility of pH affecting cell growth, pH measurements were performed to assess the potential influence of pH change on cell viability. The pH of samples was measured with the use of a pH meter (Mettler Toledo, Greifensee, Switzerland), equipped with a composite electrode (Hydromet, Gliwice, Poland, type ERH-12-6 No. 2162). The pH value was evaluated after 24, 48, and 72 h of cell culture.

### 2.6. Cell Culture

To ensure sterility and safety, all procedures regarding cell culture were carried out using a class II laminar flow cabinet (Bio II Advance—Telstar, Barcelona, Spain). Human bladder carcinoma line T24 (CLS Cell Lines Service) were cultured using DMEM/Ham’s F-12 (Corning, New York, NY, USA) while the human renal carcinoma 786-0 (American Type Culture Collection) and human renal carcinoma cell line 786-0 (American Type Culture Collection) were cultured using RPMI1640 (Corning, New York, NY, USA). Both media listed above were supplemented with 10% FBS (Corning, New York, NY, USA), 5 μg/mL amphotericin B, 100 U/mL penicillin, and 100 μg/mL streptomycin (Corning, New York, NY, USA). Cells were cultured at 37 °C in a humidified incubator under 5% of medical grade CO_2_.

### 2.7. Morphological Assessment

Morphological evaluation of cells was performed with the use of an inverted phase contrast microscope (CKX53-FL, Olympus, Tokyo, Japan) and a dedicated 4K color camera (UC90, Olympus, Tokyo, Japan). Observations were conducted for both cell lines cultured with all tested compound configurations at 24, 48, and 72 h. 

### 2.8. Viability

Cell viability was measured with the use of an MTT assay (Sigma-Aldrich, Saint Louis, MO, USA) according to ISO 10993-5:2009 guidelines. The MTT assay is a well known, commercially available, colorimetric assay for assessing cell metabolic activity (cell viability). It is based on the ability of (NADPH)-dependent cellular oxidoreductase enzymes to reduce the tetrazolium dye MTT (3-(4,5-dimethylthiazol-2-yl)-2,5-diphenyltetrazolium bromide) to its formazan salt, which was detected via a spectrophotometer.

Cells were seeded at 2 × 10^3^/well in a 96-well plate (96 flat bottom well plate, Falcon, New York, NY, USA). After a set time of exposition, cell media were removed, growth wells were rinsed with 100 μL of PBS (Corning, New York, NY, USA), and a MTT reagent solution (1 mg/mL) was added. Then, cells were incubated for 2 h at 37 °C. After incubation, the residing assay solution was discarded and the formed formazan crystals were dissolved in 100 μL DMSO (Sigma-Aldrich, Saint Louis, Missouri, USA). The absorbance was measured at 570 nm and a reference wavelength of 650 nm using a microplate reader (iMarkTM Microplate Reader BIO-RAD, Philadelphia, PA, USA).

### 2.9. Apoptosis

Cell death pathway was assessed using CellEvent™ Caspase-3/7 Green Detection Reagent (Invitrogen by Thermo Fisher Scientific, Waltham, MA, USA) according to the manufacturer’s protocol. After fixation and permeabilization, cells’ nuclei were counter-stained with aqueous DAPI solution (Cayman Chemical, Ann Arbor, MI, USA) at a concentration of 0.2 μg/mL. Visualization of apoptosis was performed using a fluorescence microscope (CKX53-FL Olympus, Tokio, Japan) and a dedicated camera (UC90 Olympus, Tokio, Japan). Results were analyzed using dedicated CellSens software (Olympus, Tokio, Japan).

### 2.10. Proliferation

Real-time proliferation analysis was performed with the xCELLigence analyzer (Roche, Basel, Switzerland). In order to determine the rate of cell proliferation, cells were cultured on a 16-plate E-Plate (Roche, Basel, Switzerland) using 4 × 10^3^ cells/well. Monitoring of cell proliferation was carried out for 84 h. 

### 2.11. Statistical Analysis

Results from at least three repeated experiments were presented as mean ± standard deviation (SD). Statistical analyses were performed using STATISTICA (StatSoft, Kraków, Poland). ANOVA analysis was used with Turkey’s post-hoc test for the performed assays with a confidence interval of 95. For the abnormal distributions, a t-test with Mann–Whitney post-test was used.

**Figure 1:**
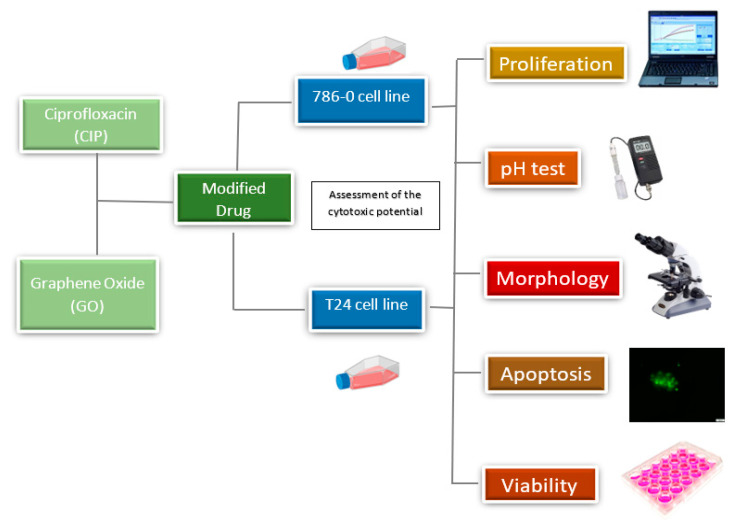
Flow diagram presenting the research methodology.

All experimental steps have been presented schematically in Figure 1.

## 3. Results

### 3.1. Graphene Oxide Characterization

**Figure 2:**
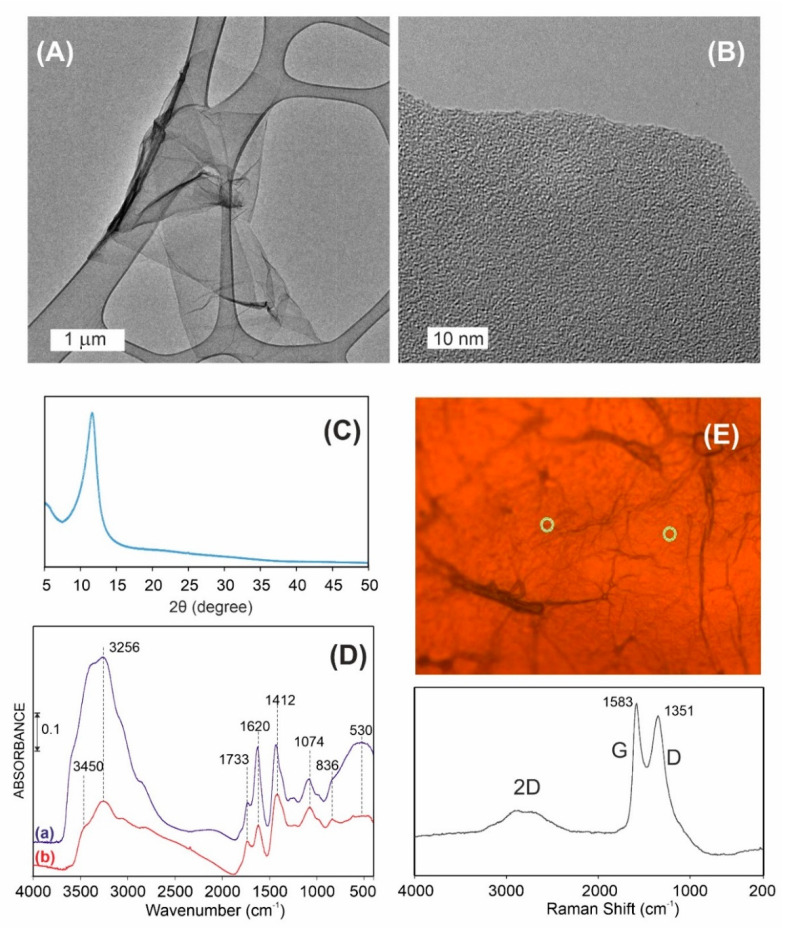
Characterization of obtained GO. The results from (**A**) and (**B**) HRTEM, (**C**) XRD, (**D**) FTIR: (a) as received, (b) after outgassing at 25 °C, and (**E**) Raman spectroscopy analysis.

GO characterization was performed with a combination of proven and innovative techniques such as high-resolution transmission electron microscopy (HRTEM—Figure 2A,B), XRD (Figure 2C), FTIR (Figure 2D), and Raman spectroscopy (Figure 2E). The results proved that GO forms large, single-layered, turbostratic structures.

In order to determine the average distance between GO layers, the XRD measurements were performed (Figure 2C). Based on this result, the only signal, appearing at 2theta ca. 11.66°, presented the calculated distance of 7.58 Å, based on Bragg’s equation.

From the Raman measurements, one can conclude that the tested sample is a typical GO with G and D bands which are characteristic of carbonaceous materials, with the maxima respectively at 1583 and 1351 cm^−1^ (Figure 2E). The broadness and low intensity of mutually overlapped 2D bands confirm structural disorder in the GO sample. This effect is due to the presence of high surface concentration of oxygen functionalities, confirmed by FTIR measurements (Figure 2D). Moreover, the obtained results showed that GO strongly interacts with water, through strong H-bonds. This is typical for highly functionalized carbonaceous structures.

### 3.2. Ciprofloxacin and Graphene Oxide Combination Characterization

**Figure 3:**
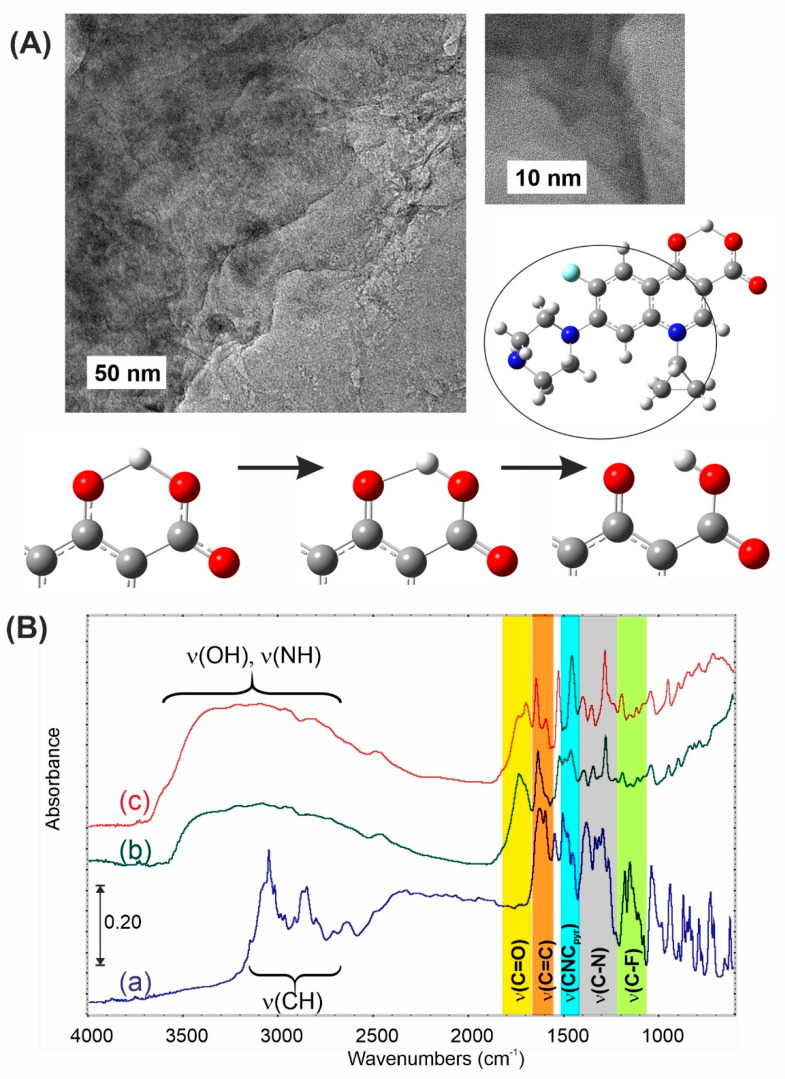
(**A**) HRTEM pictures of CIP:GO 1:1. (**B**) FTIR spectra of pure CIP: (a) and in complex with GO: (b) 1:2, (c) 1:1; with the scheme showing CIP internal salts destruction. The circle denotes the part of the drug involved in the adsorption process.

HRTEM showed no crystallinity of CIP, indicating very good interaction of the drug with the support. FTIR results confirm this statement. Figure 3 shows that there were extra peaks appearing (C=O stretching) as well as a shift in peaks’ positions when comparing the IR chart of ciprofloxacin alone with the IR chart of the mixture of CIP and GO. This is clear evidence of the detection of strong chemical interaction, probably through the N- and F-containing part. 

The most important spectral changes are observed in the C=O stretching region. While the pristine CIP forms internal salts, causing the disappearance of carbonyl bands, due to strong H-bonding, in the complex with GO, typical carboxyl and carbonyl functionalities are rebuilt (see scheme in Figure 3). This can be observed when the N-containing part of CIP molecules is involved in interactions with the GO support. The lack of v(NH) and v(CH) in the spectra (b) and (c) confirms this statement. Moreover, the pyridinic fragment of CIP was red-shifted from 1503 down to 1455 cm^−1^. This observation means that basic sites of CIP interact strongly with GO acidic functionalities. Nevertheless, in the vibrational spectra of related compounds, the bands due to C-F stretching vibrations may be found over a wide frequency range 1360–1000 cm^−1^ [23], since the vibration is easily affected by adjacent atoms and/or groups. In the present study, the FTIR bands observed at 1316 cm^−1^ have been assigned to the C-F stretching mode of vibration. 

### 3.3. Viability

**Figure 4:**
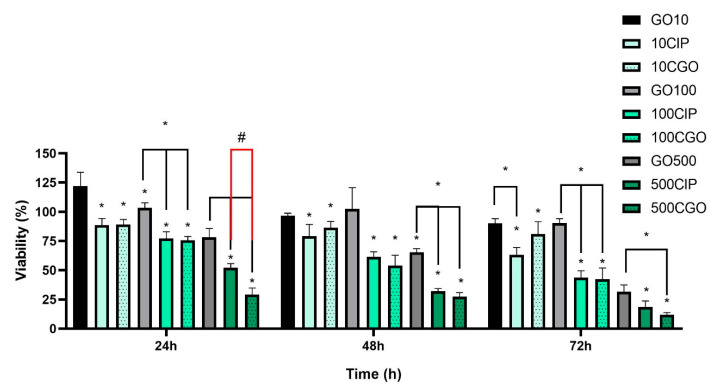
Results of the MTT assay for the T24 cell line in the following exposition times from the start of incubation with the addition of ciprofloxacin and ciprofloxacin modified from the 10–500μM concentration range. Statistically significant differences compared to the control marked as “*” above the averages on the graph. Statistically significant differences between variants marked as “*” above a clamp (*p* < 0.05). “#”—statistically significant difference between the corresponding concentrations of ciprofloxacin and modified ciprofloxacin (*p* < 0.05). An additional experiment was performed using controls that contained the solvents used with the tested compounds, i.e., deionized water and PBS at concentrations correlating to those present at the studied solutions. There was no significant difference in cell viability with those tested solvents compared to control (results not shown).

All viability results acquired via MTT assay are shown as means (±SD) of % viabilities of a given group compared to controls at specific exposition times. Statistically significant changes in viability of T24 cells were observed as soon as 24 h after exposition with all CIP and CGO concentrations. After 48 and 72 h, a further decrease in viability was observed with all tested solutions. At 24 h and 48 h, a statistically significant difference compared to control in viability was observed between CIP and CGO, of which the difference between 500 CIP and 500 CGO was the biggest (52.09 ± 3.64 vs. 29.03 ± 5.83) (Figure 4). For the T24 cell line, the results of the assay show strong cytotoxic activity of both ciprofloxacin and GO-modified ciprofloxacin. All tested concentrations of CIP and CGO caused a statistically significant decrease in cell viability compared to the control. However, for concentrations in the range of 100–500 μg/mL, a decrease was maintained for a duration of 72 h (Figure 4). A statistically significant difference in viability was observed with the addition of GO, at 500 μg/mL at 24, 48, 72 h. A statistical dependence was demonstrated between the control of graphene oxide at individual concentrations and the corresponding drug concentrations.

Results from 1000 μg/mL GO are not shown because unfortunately the GO stock solution that we started working with had too low of a concentration to prepare a 1000 μg/mL solution with our culture medium that would not falsely decrease the cell viability based on the original medium. Furthermore, we already gained sufficient results based on 10–500 μg/mL concentrations, proving the difference in cell growth kinetics and proving what we have obtained with cell morphological analysis. In both of those cases, the main mechanism of decreasing cell growth kinetics was most likely a special limitation.

**Figure 5:**
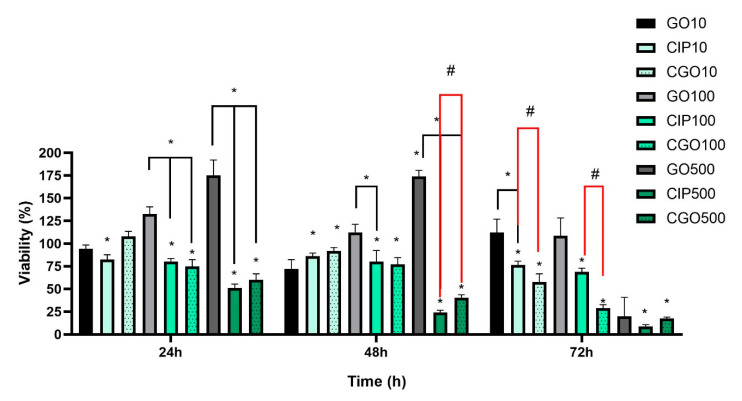
The results of the MTT test for the 786-0 line in the following days from the start of incubation with the addition of ciprofloxacin and ciprofloxacin modified from the 10–500 μM concentration range and graphene oxide in the concentration range of 10–500 μg/mL. Statistically significant differences compared to the control marked as “*” above the averages on the graph. Statistically significant differences between variants marked as “*” above a clamp (*p* < 0.05); “#”—statistically significant difference between the corresponding concentrations of ciprofloxacin and modified ciprofloxacin (*p* < 0.05).

Statistically significant changes in metabolic activity of 786-0 cells after 48 h were detected for CIP and CGO, decreasing by 74.43% (*p* < 0.05) and 57.71% (*p* < 0.05) in 500μM, respectively (Figure 5). Statistically significant changes in metabolic activity after 72 h were observed for 10μM (CIP 23.91% and CGO 41.64%) and 100 μM (CIP 25.72% and CGO 72.94%) (Figure 5).

The results of the MTT test for the control of graphene oxide in concentrations of 10 and 100 μg/mL show no statistically significant differences compared to cells cultured in an un-supplemented control medium. During the first 24 and 48 h of incubation, no statistically significant cytotoxicity effects were found for all tested GO concentrations. Statistical dependence was demonstrated between the control of graphene oxide at individual concentrations and the corresponding drug concentrations (Figure 5).

### 3.4. Proliferation

**Figure 6:**
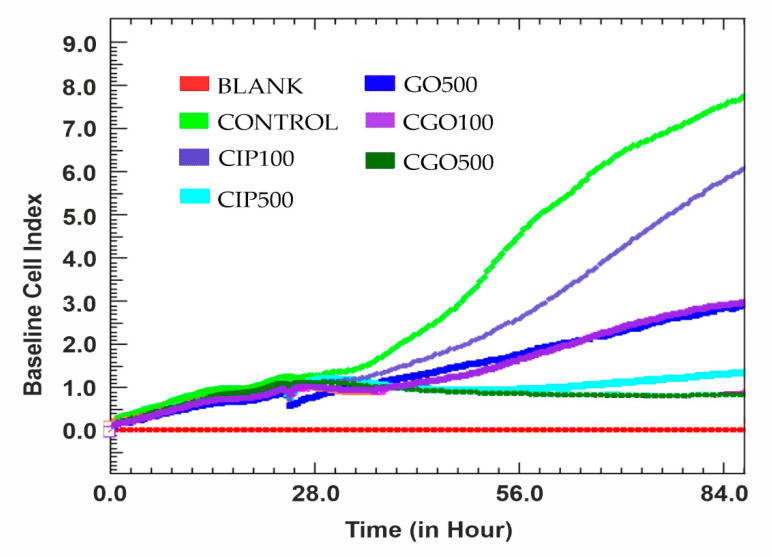
The results of the test performed using the xCELLigence analyzer for the T24 line during 84 h with the addition of ciprofloxacin and modified ciprofloxacin in the concentration range 100–500 μM. The statistical analysis was made with sampled data from specific time points. The cell index parameter is proportional to the amount of cells currently adherent to the e-plate and was measured via impedance compared to blank.

The performed analysis confirms the higher cytotoxic potential of graphene oxide-modified ciprofloxacin compared to the unmodified drug on both T24 and 786-0 cells when used in 100 and 500 μM concentrations (Figure 6). Considering T24 cells, their logarithmic growth phase was much less rapid when 500 μg/mL graphene oxide was the medium supplement; no time translation compared to the control was observed and the stationary phase occurred in a similar time. For the concentration level of 500 μM of both modified and unmodified ciprofloxacin, no logarithmic growth phase was noted. Use of 100 μM ciprofloxacin modified with GO resulted in a transition from the logarithmic phase to the 84th hour of the experiment into a rapid phase of cell death.

**Figure 7:**
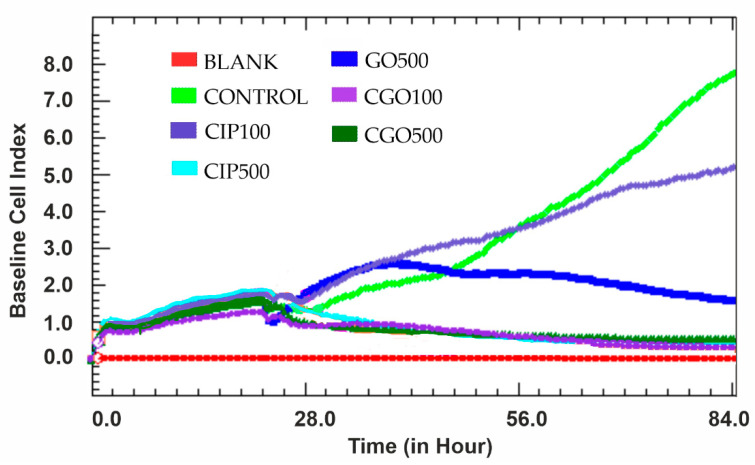
The results of the test performed using the xCELLigence analyzer for the 786-0 line during 84 h with the addition of ciprofloxacin and modified ciprofloxacin in the concentration range 100–500 μM. The statistical analysis was made with sampled data from specific time points. The cell index parameter is proportional to the amount of cells currently adherent to the e-plate and was measured via impedance compared to blank.

There is no significant difference between CGO and CIP at concentrations of 100 and 500 μM (Figure 7). The logarithmic growth phase is not observed in these concentrations. The highest cytotoxicity is with ciprofloxacin at a concentration of 1000 μM. In control cells of the 786-0 line, the logarithmic growth phase lasts up to 84 h of measurement. For the control of graphene oxide at 500 μg/mL, from the 40th hour of the experiment, there was a slow decrease in cell viability, visible until the end of the study.

### 3.5. Apoptosis

**Figure 8:**
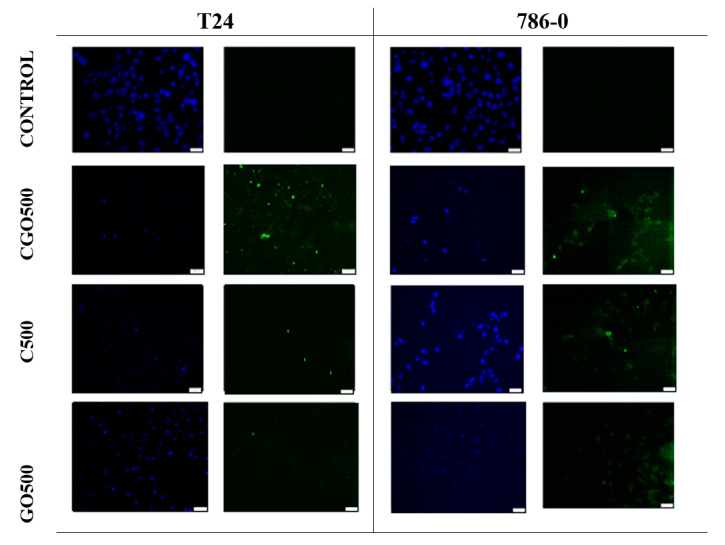
Cells of the 786-0 and T24 lines showing the results of the active caspase 3/7 assay. DAPI-stained cell nucleus, FITC—cells that underwent apoptosis. The photos were taken with the CKX53 FN Olympus fluorescence microscope, Japan, and the VC90 4 K Olympus camera, Japan. For the test samples with graphene oxide, autofluorescence of the compound itself was observed, which significantly hindered the observation of apoptotic cells. Scale bar is 50 µm.

Considering T24 cells, only single cells undergoing apoptosis were observed during the experiment. Larger clusters of apoptotic cells were observed when ciprofloxacin 500 μM was tested. In the case of graphene oxide and ciprofloxacin 500 μM, aggregates of several cells undergoing apoptosis were observed (Figure 8).

Furthermore, 786-0 cells exhibited apoptosis when ciprofloxacin 500 μM was used, while in the sample treated with modified ciprofloxacin at a concentration of 500 μM, single cells undergoing apoptosis were also present (Figure 8).

### 3.6. Morphology

**Figure 9:**
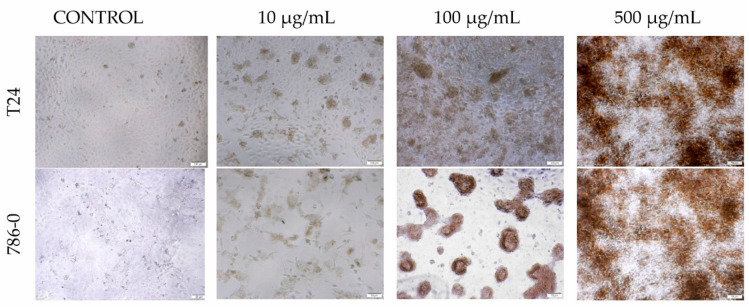
Morphology of T24 and 786-0 cells after 72 h incubation. Magnification ×50. In the case of the control group in the 10–500 μg/mL range, large dark aggregates of GO molecules are noticeable. It can limit the cell growth surface.

Morphological analysis of T24 and 786-0 cell lines after the addition of tested solutions shows a clear trend and points towards dose-dependent toxicity. The compound concentration range of 100–500 μM has proven to cause significant morphological aberrations visible at both modified and unmodified ciprofloxacin. Phase contrast microscopy proved detectable granularities around the nucleus, signs of cell detachment from the growth surface and severe cytoplasmic vacuolation (Figure 9). In the case of the control group (GO), in the 10–500 μg/mL concentration range, large dark aggregates of GO residue were noticeable and visibly were limiting the available cell growth area (Figure 9).

**Figure 10:**
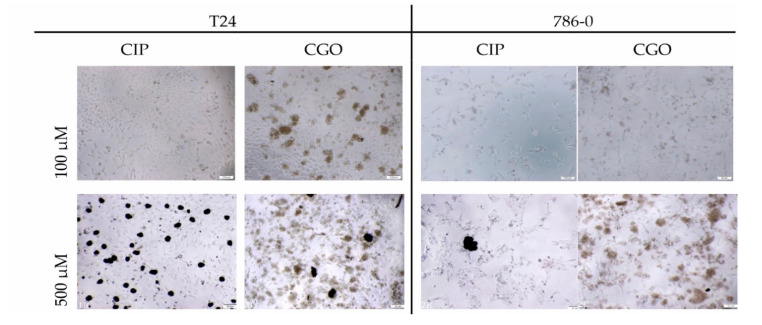
Morphology of T24 and 786-0 cells after 72 h incubation. Magnification ×50.

In the case of modified ciprofloxacin, large particles of graphene oxide are particularly noticeable, especially in the 100–500 μM concentration range, which significantly hinders the evaluation of cell morphology. However, in the case of bladder cancer line, after 72 h, at concentration of 500 μM, crystals of ciprofloxacin that precipitate are visible and likely to be released from the graphene oxide molecule chains (Figure 10).

### 3.7. Medium pH Analysis

After a 24 h incubation period, no significant difference between the pH values of all tested solutions was observed. It was found that the only supplemented media with a significantly higher pH than the control were the GO 500 μg/mL and CIP 1000 μM after 72 h incubation, the latter of which showed the highest pH of all tested solutions (Table 1).

The pH levels were presented as the mean ± SD. Interestingly, changes in the pH of the medium indicate a more alkaline reaction of the medium in which the bladder cancer line cells were grown. We took measurements of medium pH during cell culture, after 24 and 72 h of incubation. The pH values were presented as the mean ± SD.

## 4. Discussion

Studies from recent years have demonstrated the cytotoxic effect of ciprofloxacin on bladder cancer and renal adenocarcinoma cell lines [24,25]. Our results have shown that the modification of ciprofloxacin with nanomaterials such as graphene oxide may favorably increase the cytotoxicity of this chemotherapeutic agent. For the bladder cancer cell line (T24), modified ciprofloxacin showed greater mortality of cells at concentrations between 100 and 500 μM throughout the duration of the experiment; however, statistically significant differences in the action of both compounds are visible only at the highest ciprofloxacin concentration, 500 μM at 48 h, where modified ciprofloxacin causes almost complete mortality of T24 cells.

However, ciprofloxacin, often used in clinical practice and taken orally, contains extra compounds, such as HCl and lactic acid additions [26]. Thanks to these modifications, it is better absorbed by the body and can achieve higher solubility. Unfortunately, if this type of ciprofloxacin was used for in vitro studies, cell growth parameters could be affected from a significant reduction in medium pH. Therefore, it was necessary to use a modified solvent to increase the amount of dissolved ciprofloxacin and decrease the amount of solvent used. In our case, a solution of pH 4.7 was used to generate a working solution of pure ciprofloxacin. As a result, boundary solubility was obtained, which unfortunately was associated with the risk of precipitation of crystals of ciprofloxacin in the medium after addition to an assay plate. These crystals were visible at the highest concentrations of CIP under a phase contrast microscope. They were also visible after 72 h incubation as released from graphene oxide matrix at the highest concentrations of the chemotherapeutic being studied. These crystals are especially visible in the case of the T24 line, which may be connected to the faster metabolic rate of these cells, compared to the 786-0 line. In the case of renal adenocarcinoma, modified ciprofloxacin shows higher cytotoxicity towards cells only after 72 h of incubation. Microscopic observation shows that ciprofloxacin is released to a much lesser extent from graphene oxide, which may influence the more effective action of ciprofloxacin for the first 48 h.

In recent research, the drug most often paired with GO is doxorubicin. Inspired by Yang et al., in our research, we also confirm the dependence of the drug release from the pH-dependent graphene oxide molecule. As Yang’s team shows, in a neutral (pH 7) and alkaline (pH 9) environment, a chemotherapeutic—in this case, doxorubicin—was released within 80 h at 7.5% and 11%, respectively. The most effective and fastest release of the drug was demonstrated in an acidic environment (pH 5)—around 24%, which seems to be a promising aspect, due to the presence of a low pH environment inside the cancerous tumor [27]. This fact makes the combination of cytostatic with graphene oxide increase its stability, prevent the release at physiological pH, and thus reduce systematic cytotoxicity. It has also been shown that, at low pH levels (1–2), it is also not conducive to the effective release of the drug, which may be important in the case of medications administered orally because they will not be released under the outflow of the very low pH of gastric juice [28]. pH measurement for this work shows a higher pH, close to pH 9, in the case of T24 cells compared to kidney adenocarcinoma cells, where the pH was close to neutral. Research by Yang et al. [27] confirms that this may have an impact on the more effective release of the chemotherapeutic agent from graphene oxide molecules. It is also worth considering the need to increase the effectiveness of drug release, because in the absence of control over the precise combination of both compounds, it is impossible to precisely monitor this process. 

To increase the kinetics of ciprofloxacin release, Huang et al. [29] used graphene oxide layers crosslinked with a polyethyleneimine polymer. This significantly increased the durability of the conjugate, as compared to the use of graphene oxide alone, and moreover, an additional space for the placement of the drug was obtained. A molecule thus constructed was tested in vitro at various pH values, obtaining satisfactory results of the ciprofloxacin centricity release model, where pH-dependent slow drug release was demonstrated [29]. Evaluation of cell proliferation rate using the xCELLigence analyzer indicates that cells cultured with the addition of graphene oxide remain at a constant level of proliferation throughout the duration of the study. This may suggest the lack of toxic effects of graphene oxide in favor of limiting the cell growth surface occupied by large aggregates of this compound while the assay was running. 

In clinical terms, it is important to assess the effect of size and dose of graphene oxide to evaluate the distribution of the modified drug. It has been shown that, regardless of the size of the molecule, graphene oxide is quickly removed from the blood and accumulated mainly in the liver and lungs. Absorption in the lungs is greater with increasing particle size and dose concentration. The size and dose of graphene oxide affect its distribution in vivo. In microscopic image analysis, the assessment of cell morphology is difficult due to the size and color of graphene oxide molecules. They are easily noticeable in the microscopic picture and prevent cells from sharing. 

The results of the MTT test also confirm a decrease in cell viability due to the action of graphene oxide. Studies by Tyagi et al. [30] indicate a time-dependence for cells exposed to graphene oxide, where the likely cause of growth inhibition, as they describe, is the lack of access to nutrients caused by the deposition of graphene oxide aggregates on cells. As in the present study, Changa et al. [31] indicate that a significant decrease in cell survival is noticeable at higher concentrations, and Seabra and his team [32] confirm that cell viability is influenced by concentrations of graphene oxide above 100 μg/mL. 

Unfortunately, many reports have shown that nanomaterials might have side effects on health [33,34,35]. The size of GO sheets has an effect on the toxicity of GO at high concentrations and small particle sizes are recommended for medical applications [36]. Jasim and team [37] showed that, within the first hour after administration, a strong signal was observed, indicating the accumulation of graphene oxide in the bladder, spleen, and liver, and an increase in the concentration of graphene oxide in the blood. Four hours after the injection, a stronger signal was detected in the bladder, indicating further urinary excretion. This was consistent with the significant amount of graphene oxide detected in urine samples after 24 h. The shift of the liver signal activity to the spleen was observed between 4 and 24 h after administration, with signal accumulation mainly in the spleen after 24 h [37]. However, no significant signals indicating the accumulation of graphene oxide in the kidneys were detected. In addition, no organ damage or other structural changes were observed in all organs examined. However, due to the fact that chemotherapy is a long-term treatment, often lasting several months, it is equally important to assess the long-term effects of graphene oxide.

Studies performed by Wen’s team [38] involving a 6-month observation period showed that graphene oxide nanoparticles accumulate mainly in the lungs, liver, and spleen, where they persist for at least 6 months. Such long-term accumulation in tissues may have a toxic effect on these organs, hindering their proper functioning [38]. 

An important feature of graphene oxide for use in the delivery of medicines is its structure. The graphene monolayer is a structure in which each atom is exposed on the surface, allowing a significant increase in the loading capacity of drugs in comparison with other nanomaterials [39]. The appearance of the molecule also plays a very important role, especially because graphene and graphene oxide have a unique flat two-dimensional shape, which differs significantly from other known nanomaterials [40]. Among the nanoparticles, this is an exceptional shape whose model is still being described by subsequent researchers. Therefore, further research is needed on its structure and the type of death that it causes in cells. Apoptosis assay showed clearly that ciprofloxacin possesses apoptosis-inducing properties. Apoptosis was also noted for modified ciprofloxacin but at a slightly lower rate. Literature data also indicate apoptosis as the main mechanism of death of cells treated with drugs modified with graphene oxide. In a study comparing the effect of doxorubicin and doxorubicin modified with graphene oxide on human multiple myeloma cells, higher cytotoxicity of the modified chemotherapeutic agent was also demonstrated, and the main type of death of cells treated with modified doxorubicin was confirmed to be apoptosis. 

In addition, graphene oxide has been observed to cause low cytotoxicity but does not induce apoptosis and has no effect on the cell cycle [41]. Evaluation of 3/7 caspase activity allows us to conclude that graphene oxide does not induce apoptosis, which may also confirm the theory on the mechanical inhibition of cell growth. However, there are reports that graphene oxide may cause oxidative stress [39,42]. GO toxicity is obviously going to increase during higher incubation times, as described earlier. There is no clear answer as to what may cause cytotoxicity. Unfortunately, cell oxidative stress was not part of this study. It is also still unclear whether or not this aspect of GO is a drawback: some papers suggest that it is actually a desirable, anticancer trait [43]. There is even research suggesting GO as non-toxic [44]. Due to its size and attachment to the surface of cells, it is also likely that the action of graphene oxide is on the signal transduction pathways, which are initiated on the surfaces of cells [40].

Drug carriers represent a very broad topic, and the combination that we present is just one of many directions that are currently being discussed. Another very interesting candidate for a drug carrier is liposomes. Liposomes are well studied and understood, and a multitude of studies have been published on them as drug carriers. Their unquestionable advantage is the ability to modify structure—their surfaces can be modified by attaching molecules such as antibodies or antigens [45]. Coating liposomes with, e.g., polyethylene glycol inhibits liposome uptake and extends the residence time of the liposomes in the bloodstream. A characteristic feature of these long-circulating liposomal drug carriers is their increased accumulation in tumors, which is their undoubted advantage [46]. A very large barrier in the case of the use of liposomes is the high cost of lipid raw materials used to prepare the liposomes. However, the great advantage of liposomes over other carriers is the extensive knowledge of liposomes [47]. Perhaps a good way is to combine the advantages of both materials, such as wrapping of a flimsy matrix like liposome by multifunctional graphene oxide [48]. These materials have been shown to interact with each other. Scientists discovered graphene oxide-induced rupture of preabsorbed liposomes and the formation of a new composite, consisting of alternating graphene oxide monolayers and lipid membranes [49]. Perhaps this is the solution that would increase the safety of using graphene oxide in biomedical applications. We will definitely consider it in the next stages of the research.

## 5. Conclusions

Our study confirms that the modification of ciprofloxacin with graphene oxide in the treatment of urogenital tumors shows promising results with in vitro tests. However, it should be noted that this is more effective only at selected concentrations and is dependent on the type of cancer. Our results show that GO is nontoxic and inhibition of cell growth in vitro may result from limiting the growth area. Unfortunately, the risk of the accumulation of graphene oxide in organs such as the lungs or liver may be a reason to abandon the transition into the in vivo phase, because possible side effects may outweigh the advantages of using nanoparticles. We do not have a definite answer yet as to whether nanotechnology is an opportunity or a threat to modern medicine. It is necessary to look for more accurate in vitro models to assess the toxicity of nanomaterials and modified drugs.

## Figures and Tables

**Table 1:** pH medium level after 24 and 72 h cell incubation.

	786-0		T24	
	24 h	72 h	24 h	72 h
Control	7.80 ± 0.01	7.53 ± 0.01	7.90 ±0.05	7.94 ± 0.01
GO 100 μg/mL	7.91 ± 0.01	7.50 ± 0.01	8.00 ± 0.02	8.10 ± 0.01
GO 500 μg/mL	7.90 ± 0.01	7.59 ± 0.01	7.95 ± 0.03	8.09 ± 0.01
CGO 10 μM	7.87 ± 0.02	7.45 ± 0.01	7.96 ± 0.01	8.01 ± 0.01
CGO 100 μM	7.90 ± 0.01	7.51 ± 0.01	7.95 ± 0.02	8.11 ± 0.01
CGO 500 μM	7.77 ± 0.01	7.56 ± 0.02	7.88 ± 0.01	8.23 ± 0.02
CIP 10 μM	7.87 ± 0.01	7.38 ± 0.01	7.98 ± 0.03	7.95 ± 0.01
CIP 100 μM	7.81 ± 0.01	7.52 ± 0.01	8.01 ± 0.01	8.12 ± 0.02
CIP 500 μM	7.91 ± 0.01	7.70 ± 0.01	7.99 ± 0.01	8.46 ± 0.03
